# Micromixing Study of a Clustered Countercurrent-Flow Micro-Channel Reactor and Its Application in the Precipitation of Ultrafine Manganese Dioxide

**DOI:** 10.3390/mi9110549

**Published:** 2018-10-26

**Authors:** Kun-Peng Cheng, Bo Wu, Ren-Jie Gu, Li-Xiong Wen

**Affiliations:** 1 State Key Laboratory of Organic-Inorganic Composites, Beijing University of Chemical Technology, Beijing 100029, China; 2017400006@mail.buct.edu.cn (K.-P.C.); 2016410012@mail.buct.edu.cn (R.-J.G.); 2 Research Center of the Ministry of Education for High Gravity Engineering and Technology, Beijing University of Chemical Technology, Beijing 100029, China; wxmaxiaomeng@163.com

**Keywords:** clustered countercurrent-flow micro-channel reactor, micromixing time, preparation, ultrafine particle, manganese dioxide

## Abstract

A clustered countercurrent-flow micro-channel reactor (C-CFMCR) has been assembled by the numbering-up of its single counterpart (S-CFMCR). Its micromixing performance was then studied experimentally using a competitive parallel reaction system, and the micromixing time was calculated as the micromixing performance index. It was found that the micromixing time of C-CFMCR was ranged from 0.34 to 10 ms according to its numbering-up times and the operating conditions of the reactor, and it was close to that of S-CFMCR under the same operating conditions, demonstrating a weak scaling-up effect from S-CFMCR to C-CFMCR. The C-CFMCR was then applied to prepare ultrafine manganese dioxide in a continuous manner at varying micromixing time. It showed that the micromixing time had a major effect on the particle structure. More uniform and smaller MnO_2_ particles were obtained with intensified micromixing. By building a typical three electrode system to characterize their performance as a supercapacitor material, the MnO_2_ particles prepared by both S-CFMCR and C-CFMCR under optimal conditions displayed a specific capacitance of ~175 F·g^−1^ at the current density of 1 A·g^−1^, with a decline of ~10% after 500 charge-discharge cycles. This work showed that C-CFMCR will have a great potential for the continuous and large-scale preparation of ultrafine particles.

## 1. Introduction

Precipitation is an extensive process in chemical industries for the preparation of particles of various size distributions, morphologies and other properties, in which the achievement of supersaturation—thermodynamically controlled phase transition—is significant because of the highly nonlinear dependency of both nucleation and growth rates on supersaturation [1]. As precipitation is generally associated with very fast reactions, the mixing behavior of different reacting materials is essential for guaranteeing the quality and stability of the product. The complex mixing process can be described as three simpler stages, i.e., macromixing (mixing on the scale of vessel), mesomixing (mixing on the scale of turbulent eddies) and micromixing (mixing on molecular scale, with viscous-convective deformation and diffusion) [2]. Micromixing is among the most important process which is likely to promote the uniform level of high supersaturation, and hence the product properties. Consequently, fast micromixing is desired to generate supersaturation through mixing processes. In fact, the characteristic micromixing time scale must be low enough to be theoretically able to compete with the characteristic reaction time scale as well as the precipitation time scale [3].

Therefore, the select of mixing technology is particularly important. However, it is difficult to achieve a fast micromixing in the most commonly used stirred tank reactors due to the inhomogeneous distribution of specific power dissipated. Many devices for strengthening the micromixing performance have been developed, such as confined impinging jets reactor [4], rotor-stator mixer [5], Y-type micro-channel reactor [6], microporous tube-in-tube micro-channel reactor [7], etc. However, all of these equipment suffer from two important drawbacks: Most of them are difficult to manufacture and scale up due to their complicated structures; and, as to precipitation processes, low throughput and severe blocking problems have greatly restricted their applications [8,9], which allows only small-scale production.

Pseudocapacitive materials, such as transition metal and conducting polymers, have been of great interest due to their practical applications in the electronics industry [10,11]. Among those materials, manganese dioxide (MnO_2_) with the advantages of low cost and sufficient electrochemical activity, has attracted increasing attention as active electrode materials [12,13]. MnO_2_ exists in a variety of crystal structures (*α*-, *β*-, *γ*-, and *δ*-), and *α*-MnO_2_ has the highest theoretical specific capacitance due to its tunnel structures [14]. Research has indicated that a decreased particles size of MnO_2_ with an enlarged specific surface area can supply more active sites and shorten the diffusion time of electrons and ions, thus improving its capacitive performance [15,16]. There are many methods for the synthesis of ultrafine *α*-MnO_2_, such as reaction precipitation [17], hydrothermal [18,19,20], sol-gel [21], micro-emulsion [22], pyrolysis method [23], etc. Compared to other methods, reaction precipitation offers many advantages such as a flexible and simple process under mild conditions with a short-term reaction time. Cheney et al. [24] proposed that the characteristic reaction time in the MnO_2_ precipitation process is at the level of several milliseconds. Therefore, a micromixing intensified technique is needed to achieve a homogeneous nucleation environment and better supersaturation control.

In this work, a clustered countercurrent-flow micro-channel reactor (C-CFMCR) was assembled by numbering-up of its single counterpart (S-CFMCR). Micromixing performance (expressed as micromixing time) of C-CFMCR was characterized by applying iodide-iodate reaction system and the incorporation model. The C-CFMCR was then applied for the precipitation of ultrafine MnO_2_ at varying micromixing time and the performance of the prepared MnO_2_ as a supercapacitor material was evaluated by building a typical three electrode system, hence the influence of micromixing time on the particle properties will be assessed.

## 2. Experimental

### 2.1. Clustered Countercurrent-Flow Micro-Channel Reactor

**Figure 1:**
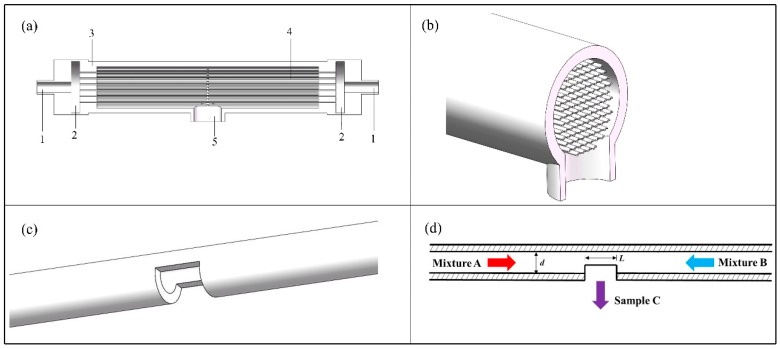
C-CFMCR: (**a**) appearance of C-CFMCR: 1. main inlet; 2. buffer zone; 3. shell; 4. S-CFMCRs; 5. main outlet; (**b**) middle cross-section of C-CFMCR.; (**c**) S-CFMCR; (**d**) schematic illustration of S-CFMCR.

The C-CFMCR (Figure 1a,b) was designed based on its counterpart, i.e., single countercurrent-flow micro-channel reactor (S-CFMCR) (Figure 1c,d). It consists of a varible number of S-CFMCR, which is made by steel micro-capillary and has a laser drilled rectangular hole in the middle. The diameter *d* and the outlet length *L* of S-CFMCR can be adjusted between 0.2 and 1 mm. These S-CFMCRs are arranged with designed patterns in a partially sealed transparent cylindrical shell. Both ends of the S-CFMCRs are integrated into two cavities at the two ends of the shell, respectively, to share the incoming reactant stream. The cavities act as buffer zones and are formed between the end of the shell and the connecting blind flange, which is also assembled with the main inlet pipe. The cylindrical shell is made of ploymethyl methacrylate for the purpose of visualization. Two reactant streams are injected into the two cavities through the main inlet, and then split and flow into all S-CFMCRs, respectively, which will collide with each other at the centering outlet of each individual S-CFMCRs to achieve intensified micromixing. The converged fluid will then flow inside the shell for further mixing and eventually out of the reactor through an outlet at the bottom of the shell. The numbering-up of S-CFMCR was thus achieved by the integration assembly of C-CFMCR, which has the advantages of easy fabrication and convenient operation. In addition, the fluid velocity within each individual S-CFMCRs can be very low since the two fluids mix mainly by convective mixing instead of intense impact, which allows a bigger magnification scale for larger productivity in consideration of the capacity of pumps.

### 2.2. Micromixing Characterization

#### 2.2.1. Experimental Test Reaction

The Villermaux-Dushman parallel competing reaction system with reported experimental procedure and reaction kinetics [25,26], was implemented in this work to characterize the reactor micromixing performance. It consists of an acid-base neutralization reaction competing with the Dushman reaction for the limiting amount of acid source. Firstly proposed by Fournier et al. [27], the exact kinetics of the system has gained a lot of research attention so as to determine the optimal choice of concentrations and operating conditions. It offers great potential to determine micromixing time as well as mixing performance as a function of process conditions in chemical reactors.

The reaction formulas are shown as follows:

(1) $$H_{2}{\mathrm{BO}}_{3}{}^{-}+H^{+}\to H_{3}{\mathrm{BO}}_{3}\;(\text{quasi-instantaneous})$$

(2) $${5I}^{-}+{\mathrm{IO}}_{3}{}^{-}+{6H}^{+}\to {3I}_{2}+{3H}_{2}O\;(\mathrm{very}\;\mathrm{fast})$$

(3) $$I^{-}+I_{2}^{}\overset{}{↔}I_{3}^{-}$$

reaction (1) is quasi-instantaneous, and the kinetics can be expressed as [28]:

*r*_1_ = *k*_1_[H^+^] [H_2_BO_3_^−^]

where *k*_1_ = 10^11^ m^3^·kmol^−1^·s^−^^1^ at 25 °C.

For reaction (2), the kinetics is experimentally determined and can be written as [26]:

*r*_2_ = *k*_2_[IO_3_^−^][I^−^]^2^[H^+^]^2^, where the kinetic rate law depends on the ionic strength *I*:

(4) $$I<0.166\;\mathrm{mol}\cdot L{}^{-1},\;\log_{10}(k_{2})=9.28-3.66\sqrt{I}$$

(5) $$I>0.166\;\mathrm{mol}\cdot L{}^{-1},\;\log_{10}(k_{2})=8.38-1.51\sqrt{I}+0.23I$$

reaction (3) is a reversible reaction whose kinetics can be specified as [26]:

*r*_3_ = *k*_3_[I^−^][I_2_] − *k*_3_’[I_3_^−^]

with *k*_3_ = 5.9 × 10^9^ m^3^·kmol^−1^·s^−^^1^ and *k*_3_’ = 7.5 × 10^9^ s^−1^ at 25 °C.

Reaction (1) is quasi-instantaneous while reaction (2) is very fast but infinitely slower than reaction (1). The two reactions are competing for H^+^ in the system. In the case of ideal micromixing, H^+^ will be instantaneously distributed homogeneously and completely consumed by borate ions (H_2_BO_3_^−^) to form boric acid (H_3_BO_3_) according to reactions (1), and (2) will not take place. On the contrary, H^+^ will be involved in both reactions (1) and (2); hence I_2_ will be formed. The formed I_2_ can further react with I^−^ to yield I_3_^−^ according to reaction (3). The amount of the produced I_3_^−^ depends on the micromixing performance, which is expressed in terms of segregation index (*X*_S_), and calculated from the following expressions:

(6) $$X_{S}=\frac{Y}{Y_{ST}}$$

where *Y* is the molar ratio of the acid consumed by reaction (2) to the total acid injected; *Y_ST_* is the value of *Y* in case of total segregation, as follows:

(7) $$Y=\frac{2\left(V_{A}+V_{B}\right)\left(\left[I_{2}]+[I_{3}^{-}\right]\right)}{V_{A}{\left[H^{+}\right]}_{0}}$$

(8) $$Y_{ST}=\frac{6{\left[{\mathrm{IO}}_{3}^{-}\right]}_{0}}{6{\left[{\mathrm{IO}}_{3}^{-}\right]}_{0}+{\left[H_{2}{\mathrm{BO}}_{3}^{-}\right]}_{0}}$$

in which, the subscript 0 represents the initial concentration of each component before entering the mixing zone; *V*_A_ and *V*_B_ denote the volumetric flow rates of mixtures A and B, respectively. In this study, mixture A is composed of H_2_BO_3_^−^, I^−^ and IO_3_^−^, and mixture B is acid solution.

The value of *X*_S_ is within the range of 0 < *X*_S_ < 1 for partial segregation. The higher *X*_S_ is, the worse the micromixing status it represents.

#### 2.2.2. Determination of the Acid and Initial Concentrations

Although sulfuric acid is typically used in characterizing micromixing efficiency associated with Villermaux-Dushman reaction system, there is a non-negligible factor related to it when referring to quantitative analysis of micromixng time. The second proton is not ionized as complete as the first one (*pK_a_*_1_ = −3, *pK_a_*_2_ = 1.99), making the hydrogen ion concentration lower than that predicted. Therefore, we chose perchloric acid instead of sulfuric acid as hydrion supplier for it is a strong monoprotic acid (*pK_a_*_1_ = −9.24) [29]. The used reactants, their initial concentrations are shown in Table 1. All reagents used were obtained from Beijing Tongguang Fine Chemicals Company (Beijing, China), and were of analytical grade and used without further purification.

For preparation of the base solution, sodium hydroxide (NaOH) was added into boric acid solution (H_3_BO_3_) in deionized water to obtain an equimolar buffer solution of H_2_BO_3_^−^/H_3_BO_3_ (pH = 9.14). Additionally, KI and KIO_3_ were dissolved in deionized water to form a KI/KIO_3_ solution, then added to the buffer solution. This sequence of mixing must be strictly followed so that iodide and iodate ions can coexist in a basic solution, which prevents thermodynamic iodine formation [25].

#### 2.2.3. Presentation of the Incorporation Model

The incorporation model was firstly proposed for estimating the micromixing time [27], especially in continuous reactors [30]. Based on the incorporation model, mixture A is divided into aggregates, which are progressively invaded by fluid of the outside mixture B. The characteristic reaction time of incorporation (*t_m_*) is assumed to be equal to the micromixing time. The volume of the aggregate grows according to the law of *Q*_2_ = *Q*_20_ *g*(*t*), in which *g*(*t*) is the incorporation function with the form of a linear relationship of *g*(*t*) = 1 + *t*/*t_m_* or an exponential model of *g*(*t*) = exp(*t*/*t_m_*). A simple dilution-reaction scheme was assumed as stated in the following equation:

(9) $$\frac{dC_{j}}{dt}=(C_{j10}-C_{j})\frac{1}{g}\frac{dg}{dt}+R_{j}$$

where *C_j_* denotes the concentration of reactant *j*, *C_j_*_10_ represents the concentration of surrounding liquid, *R_j_* is the reaction term, and *g* denotes a function controlling the mass exchange rate between the liquid particle and the surrounding liquid. The equations were solved by the Runge-Kutta method using Matlab.

### 2.3. Synthesis of Manganese Dioxide

Precipitation processes were carried out at room temperature. A manganese sulfate solution (*C*_1_ = 0.15 mol·L^−1^) and a potassium permanganate solution (*C*_2_ = 0.1 mol·L^−1^) were injected into C-CFMCR simultaneously from the two ends by using two precise plunger pump (TBT1T02, TAUTO of Shanghai) and mixed with each other, forming manganese dioxide according to Equation (10). The pipe and pump heads of the plunger pumps are made of 316 L stainless steel. The pumps possess a flowrate measurement error below 5%, with a working pressure ranging from 0 to 2 MPa. Connected with C-CFMCR by threaded structure under airtight condition, the plunger pumps could convey reactants precisely at specified volumetric flow rate into C-CFMCR with uniform distribution of fluid in each individual S-CFMCR.

(10) $$3{\mathrm{MnSO}}_{4}{+2\mathrm{KMnO}}_{4}{+2H}_{2}O\to {5\mathrm{MnO}}_{2}↓{+K}_{2}{\mathrm{SO}}_{4}{+2H}_{2}{\mathrm{SO}}_{4}$$

The volumetric flow ratio (*R*) of the two streams was kept as 1 and can be adjusted along with the concentration of reactants, with varying volumetric flow rates ranging from 100 to 1000 mL·min^−1^ with a practical working pressure of pumps varying from 0.05 to 1 MPa. The precipitate suspension flowing out of the C-CFMCR shell was collected into a stirred container and further stirred at room temperature for 0.5 h. The suspension was then filtered, washed several times and dried at 80 °C for 24 h. After grinding, the sample was calcined at 220 °C for 2 h.

### 2.4. Characterization Methods

For micromixing characterization, the absorbance of triiodide ion was measured using spectrophotometry (Shimadzu UV-2550, Kyoto, Japan) at a wave length of 353 nm. The concentration of triiodide ion was calculated according to the law of Lambert-Beer with a molar absorption coefficient experimentally determined as 2671 m^2^·mol^−^^1^.

Powder X-ray diffraction (XRD) analysis was conducted with a Bruker D8 Advance X-ray diffractometer (Bruker Corp., Berlin, Germany), using Cu K*α* radiation. Diffraction intensities were recorded by scanning from 10 to 80° (2θ).

The nitrogen adsorption-desorption isotherms were carried out using a Micromeritics ASAP 2010 adsorption apparatus (Micromeritics Instrument Corp., Norcross, GA, USA). The specific surface area (*S*_BET_) and the pore size distribution (PSD) were calculated by the BET (Brunauer-Emmett-Teller) method and BJH (Barrett-Joiner-Halenda) model, respectively.

The electrochemical performance was obtained by galvanostatic charge-discharge (GCD) measurement in a three-electrode configuration system by an electrochemical workstation (CH Instruments Inc., Austin, TX, USA). Na_2_SO_4_ solution with a concentration of 0.5 M was used as electrolyte.

## 3. Result and Discussion

### 3.1. Investigation of Micromixing Performance of C-CFMCR

#### 3.1.1. Estimation of Micromixing Time

**Figure 2:**
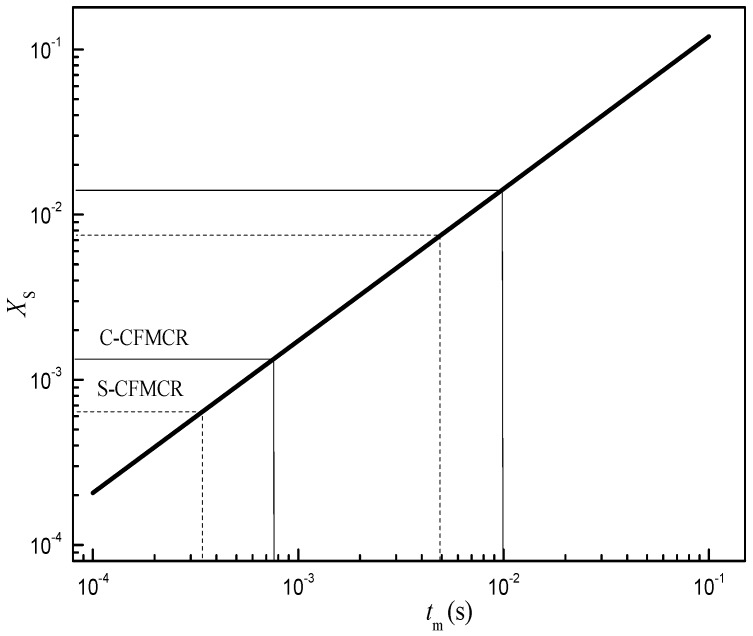
Relationship between micromixing time and segregation index.

Micromixing by incorporation model was formulated in Section 2.2.3. The mass conservation and the reaction kinetics of Villermaux-Dushman method were adapted into the incorporation model to obtain the relationship between micromixing time *t_m_* and segregation index *X*_S_ at specified experimental conditions. The fitting result of the dependency relationship between *t_m_* and *X*_S_ is given in Figure 2. In order to assure sufficient accuracy, only the log-linear range is considered for the correlation of micromixing time. In the range of 0.0001 < *X*_S_ < 0.1, where micromixing dominates the mixing process, it was found that

(11) $$X_{S}=0.99t_{m}{}^{0.92}$$

Therefore, under the studying operation conditions, the micromixing time for S-CFMCR and C-CFMCR were in the range of 0.34–4.96 ms and 0.76–10 ms, respectively.

#### 3.1.2. Influence of Volumetric Flow Rate on Micromixing Time

**Figure 3:**
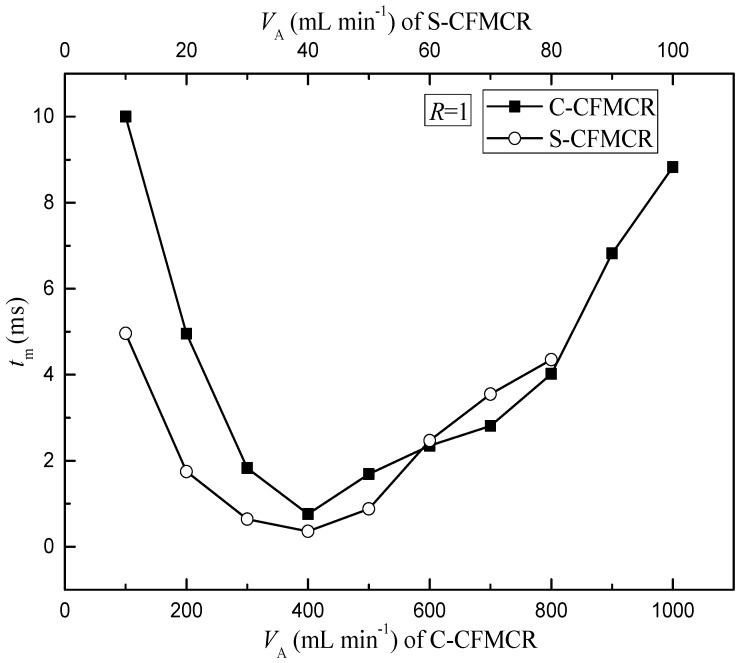
Estimated micromixing time at varying inlet volumetric flow rate.

To better evaluate the micromixing performance of the reactor and understand its applications in synthesis, a comparison of micromixing time between a typical C-CFMCR that consists of ten S-CFMCRs and its counterpart, i.e., a separate S-CFMCR, was performed at varying operation conditions. As illustrated in Figure 3, the micromixing time *t_m_* of both S-CFMCR and C-CFMCR decreased and then increased with increasing volumetric flow rate *V*_A_ with a minimum value reached at *V*_A_ = 40 and 400 min·L^−1^, respectively. The mixing within the reactors occurs mainly in the converging region of the two liquid streams. When the volumetric flow rate was too low, the flow of the two streams would not be strong enough to achieve the desired quick micromixing and result in big micromixing time; especially for C-CFMCR, the pressure within the cavity at the two ends might not be strong enough to assure even distribution of the incoming stream into the integrated ten S-CFMCRs, leading to even bigger overall micromixing time, as shown by the big gap of micromixing time between S-CFMCR and C-CFMCR at low volumetric flow rate. On the other hand, if the volumetric flow rate was too high, the flow of the two streams would then be too strong and part of the reactants would sputter out of the reactor outlet without colliding into each other, resulting in lower micromixing efficiency and a longer micromixing time. Therefore, the volumetric flow rate of 40 min·L^−1^ in each individual micro-channel was chosen as the optimal volumetric flow rate for the subsequent precipitation processes.

### 3.2. Experimental Study of Precipitation of Manganese Dioxide

#### 3.2.1. Material Characteristics

Manganese dioxide was precipitated in one S-CFMCR and two C-CFMCR with different number of integrated S-CFMCRs, respectively, under different fluid flow rates, which then possessed different micromixing time (see Table 2). It has been found in previous study that the concentration of reactants within the investigated range had almost no impact on properties of the produced MnO_2_; hence the reactant concentration was only adjusted according to volumetric flow ratio (*R*) between the injected reactant streams for assuring a constant reactant mole ratio.

**Figure 4:**
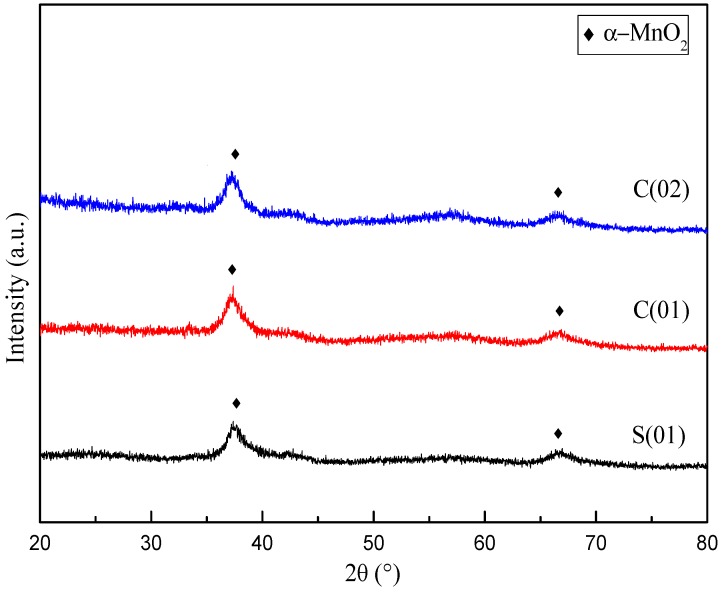
XRD patterns of MnO_2_ prepared at different operating conditions (Note: Please refer to the reactor Ref. # in Table 2).

**Figure 5:**
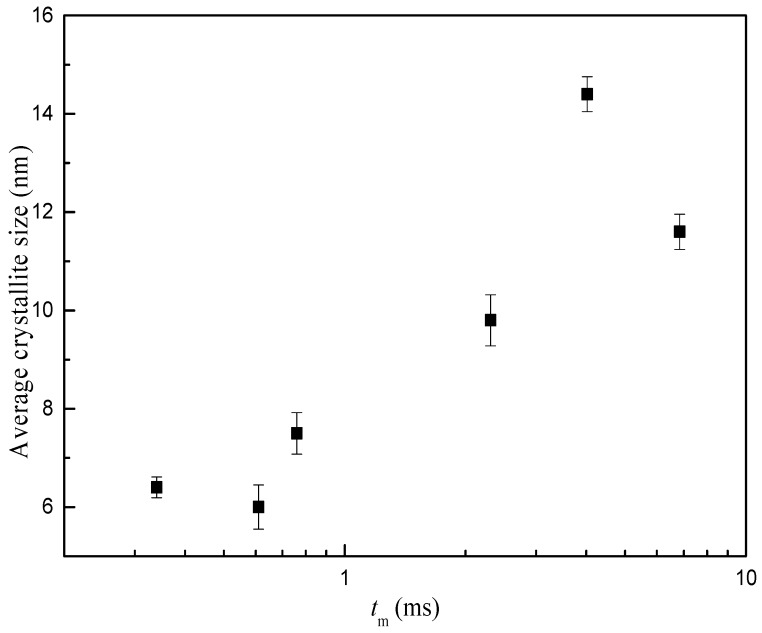
Average crystallite size variation with micromixing time.

Figure 4 demonstrates the XRD profiles for samples prepared under different operating conditions. There is no obvious distinction in XRD patterns for MnO_2_ prepared with different magnification of C-CFMCR. Unsharp and broadening peaks indicate an amorphous state. The peaks at 2θ = 37.0° and 66.5° correspond to some peaks of *α*-MnO_2_ with a tunnel structure matching JCPDS card No. 44-0141. Average crystallite size was calculated by employing Scherrer’s equation using the crystal face (2 1 1) diffraction. The variation of mean crystallite size with micromixing time is illustrated in Figure 5. Overall, increasing micromixing time would enlarge the crystallite size. The mean crystallite size varied from 6 to 8 nm at micromixing time lower than 1 ms. It gradually rose to larger than 10 nm with bigger micromixing time (*t_m_* > 1 ms). Theoretically, a reduction of micromixing time would result in a more uniform and higher level of supersaturation, which is associated with a more homogeneous nucleation process, and smaller crystallites size as a consequence. However, the experimental results in Figure 5 were not completely in agreement with the theoretical prediction, probably because that the samples shown in Figure 5 were prepared in three different reactors and also at different volumetric flow ratio for the same reactor, as listed in Table 2.

**Figure 6:**
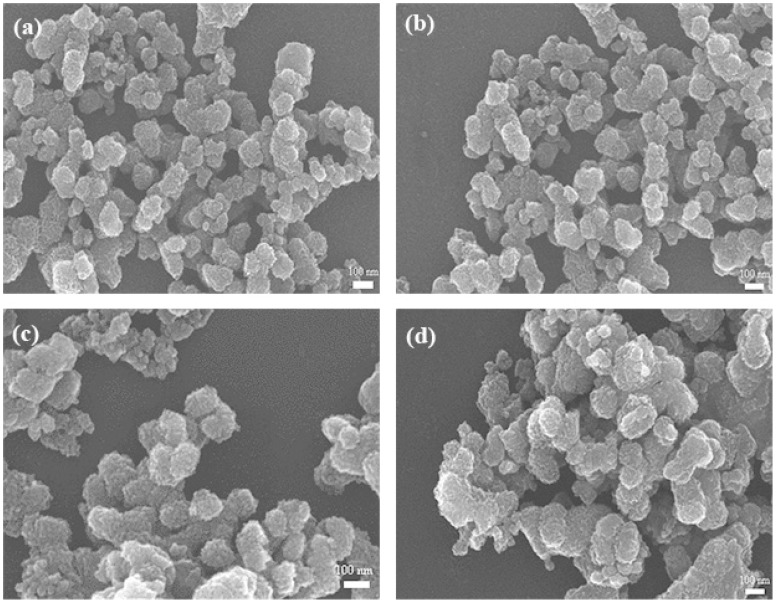
SEM images of MnO_2_ samples prepared under different conditions: (**a**) S(01); (**b**) C(01); (**c**) C(02); (**d**) C(03).

Figure 6 presents SEM images of the MnO_2_ prepared under different conditions with varying micromixing time. In accordance with the crystallite size, the S(01), C(01) and C(02) particles showed similar morphology and uniform size of ~120 nm (Figure 6a–c), due to their close micromixing time. Slightly larger and more aggregated particles were obtained with an increased micromixing time (Figure 6d). Such features may be ascribed to the different micromixing performance and levels of supersaturation during the precipitation processes. The intensified micromixing behavior (expressed as smaller micromixing time) can promote the uniform level of high supersaturation, which favors a higher primary nucleation rate, and hence result in smaller and more uniform particles.

**Figure 7:**
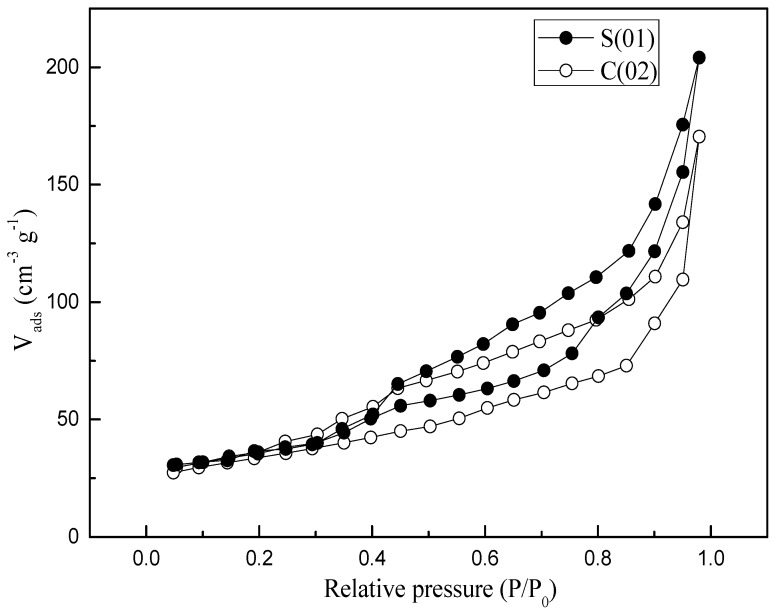
N_2_ adsorption/desorption isotherms of MnO_2_ obtained in S-CFMCR and C-CFMCR, respectively.

**Figure 8:**
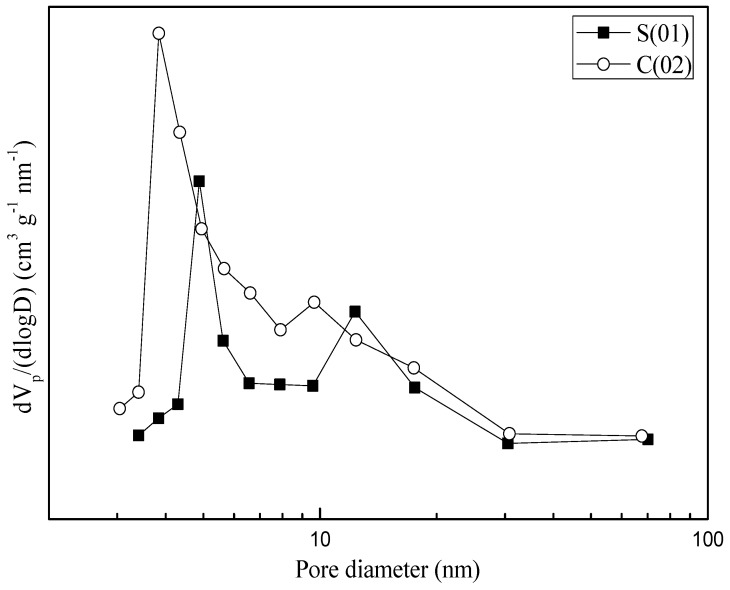
Pore size distributions of MnO_2_ obtained in S-CFMCR and C-CFMCR, respectively.

N_2_ adsorption and desorption studies were performed to assess the textural properties of the samples (see Table 3). Both samples prepared in S-CFMCR and C-CFMCR showed a type IV isotherm (see Figure 7). Pore size distributions (PSDs) of the precipitated MnO_2_ through S-CFMCR and the tenfold magnified C-CFMCR, respectively, are presented in Figure 8. Both precipitates exhibited bimodal distributions with two maxima at about 3–5 and 9–13 nm for pore diameter. The former peak may be ascribed to the small pores within the particles, which have a mean particle size of ~120 nm; the latter probably comes from the interspace among the aggregated particles. According to the IUPAC nomenclature, micropores, mesopores and macropores are representing pores less than 2 nm, between 2 and 50 nm, and greater than 50 nm in diameter, respectively. Therefore, the prepared MnO_2_ possesses a mesoporous structure with a small pore size.

Table 3 also presents the effects of micromixing time on the BET surface area of MnO_2_. There was no clear relationship between the specific surface area and micromixing time, mostly because that the samples were obtained in three different reactors under different operating conditions (see Table 2). However, Table 3 still shows that *S*_BET_ of S(01), C(01) and C(02) were very close, in which the average volumetric flow rates in each individual micro-channel were all set to 40 mL·min^−1^ and their micromixing time were close as well, demonstrating the significant effects of micromixing performance on the specific area of the produced particles. The slightly smaller *S*_BET_ of C(01) and C(02) might indicate a slight negative scaling-up effect of C-CFMCR.

#### 3.2.2. Electrochemical Properties

**Figure 9:**
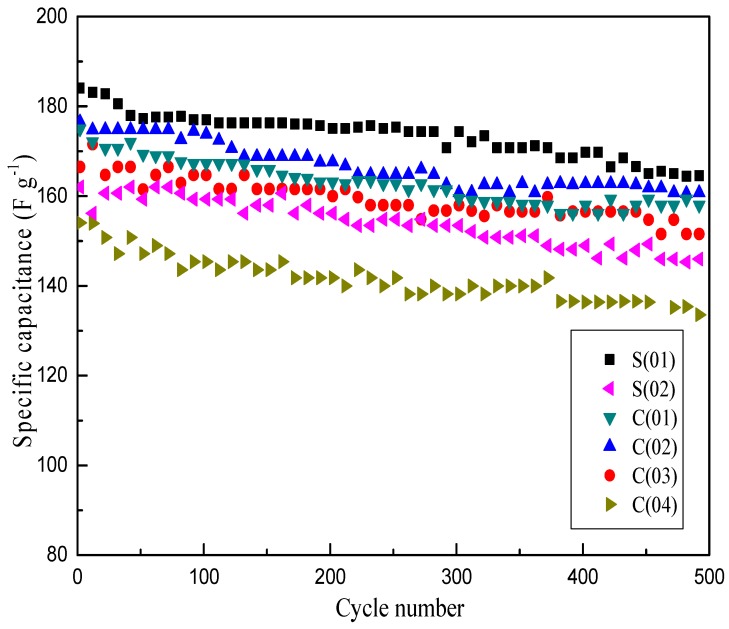
Cycle-life of MnO_2_ prepared under different operating conditions at 1 A·g^−1^.

Previous results of textural properties indicated that the prepared *α*-MnO_2_ possesses a mesoporous structure. When applied as a supercapacitor material, the transport and insertion of cations from the electrolyte are involved in the charge storage process of MnO_2_ electrode, in which well-structured material will guarantee that its most surface are directly accessible for cations [31]. Therefore, the specific capacitance (SC) is closely related to the specific surface area and crystallite size of the material. Figure 9 presents the comparison of charge-discharge cycle performance of MnO_2_ prepared under different operating conditions at a current density of 1 A·g^−1^. It shows that the SC difference between S(01) and C(02) is slight, and the maximum of SC of S(01) is just 9 F·g^−1^ higher than that of C(02), which is a C-CFMCR with ten times integrated S-CFMCR, indicating a weak scaling-up effect of S-CFMCR in the precipitation of ultrafine powder. Samples obtained at bigger volumetric flow ratios showed relatively poor SC, which could be ascribed to the weakened micromixing efficiency. Thus, equal volumetric flow rates of the two streams should be kept in the precipitation applications of C-CFMCR. Overall, samples obtained at intensified micromixing showed an improved electrochemical performance, which agreed well with the effects of micromixing on the crystallite size of the material, with smaller crystallite size favoring the redox reactions for enhanced electrochemical performance. After 500 cycles, only 10% decrease of SC was observed, which indicates stable cycling properties of the prepared MnO_2_. This result confirmed the feasibility and validity of C-CFMCR for large-scale controllable preparation of ultrafine MnO_2_ used as a supercapacitor material.

## 4. Conclusions

This study focused on the precipitation of manganese dioxide, a promising electrode material for supercapacitors. The electrochemical capacitance of manganese oxide-based capacitor is largely dependent on its textural properties. Investigations on micromixing efficiency of the reactors were thus performed, which is associated with the subsequently nucleation and aggregation processes. A clustered countercurrent-flow micro-channel reactor (C-CFMCR) was assembled by numbering-up of its single counterpart (S-CFMCR) at adjustable magnifications. Their micromixing efficiency was studied by using the Villermaux-Dushman method. The micromixing performance was controlled by adjusting the operating conditions in a continuous manner. The major findings are as follows:

Under the studied operating conditions, the micromixing time for the S-CFMCR and C-CFMCR was in the range of 0.34–4.96 ms and 0.76–10 ms, respectively, and the quickest micromixing was achieved when the average volumetric flow rate was set to 40 mL·min^−1^ in each individual micro-channel for both S-CFMCR and C-CFMCR. Both reactors could produce ultrafine MnO_2_ particles possessing a mesoporous structure with narrow pore size distribution. Overall, the intensification of micromixing time would result in smaller crystallite size and eventually larger specific capacitance of the produced MnO_2_ particles as electrochemical supercapacitor electrodes. Only a very slight amplification effect of S-CFMCR to C-CFMCR on its application in precipitation of MnO_2_ was noticed, demonstrating that C-CFMCR might be an efficient method for large-scale precipitation of ultrafine powders.

## Figures and Tables

**Table 1:** Initial concentrations of used reactant.

Reactants	Initial Concentrations (mol·L^−1^)
H_3_BO_3_	0.1818
NaOH	0.0909
KI	0.01167
KIO_3_	0.00233
HClO_4_	0.05

**Table 2:** Operating parameters for MnO_2_ precipitation in S-CFMCR and C-CFMCR.

Ref.	*C*_1_ (mol·L^−1^)	*C*_2_ (mol·L^−1^)	*R*_2/1_	*Q*_1_ (mL·min^−1^)	*Q*_2_ (mL·min^−1^)	Number of S-CFMCR	*t_m_* (ms)
S(01)	0.15	0.1	1	40	40	1	0.34
S(02)	0.3	0.1	2	20	40	1	2.31
C(01)	0.15	0.1	1	200	200	5	0.61
C(02)	0.15	0.1	1	400	400	10	0.76
C(03)	0.15	0.1	1	800	800	10	4.02
C(04)	0.3	0.1	2	200	400	10	6.84

**Table 3:** BET surface area of MnO_2_ precipitated in varying types of C-CFMCR.

Ref.	*t_m_* (ms)	*S*_BET_ (m^2^·g^−1^)
S(01)	0.34	176
S(02)	2.31	193
C(01)	0.61	154
C(02)	0.76	158
C(03)	4.02	214
C(04)	6.84	186

(Note: Please refer to the reactor Ref. # in Table 2).

## Nomenclature

*C_i_*	concentration of *i* (mol·L^−1^)
*I*	ionic strength (mol·L^−1^)
*Q_1_*	volume flow rate of manganese sulfate solution (mL·min^−1^)
*Q_2_*	volume flow rate of potassium permanganate solution (mL·min^−1^)
*R*	volumetric flow ratio
*t_m_*	micromixing time (ms)
*V_A_*	volume flow rate of iodide mixture (mL·min^−1^)
*V_B_*	volume flow rate of acid solution (mL·min^−1^)
*V_p_*	pore volume (cm^3^·g^−1^)
*X_S_*	segregation index
*Y*	selectivity of iodide
*Y_ST_*	selectivity of iodide for total segregation
